# Metabolic convergence on lipogenesis in RAS, BCR-ABL, and MYC-driven lymphoid malignancies

**DOI:** 10.1186/s40170-021-00263-8

**Published:** 2021-08-16

**Authors:** Daniel F. Liefwalker, Meital Ryan, Zhichao Wang, Khyatiben V. Pathak, Seema Plaisier, Vidhi Shah, Bobby Babra, Gabrielle S. Dewson, Ian K. Lai, Adriane R. Mosley, Patrick T. Fueger, Stephanie C. Casey, Lei Jiang, Patrick Pirrotte, Srividya Swaminathan, Rosalie C. Sears

**Affiliations:** 1 Department of Molecular and Medical Genetics, Oregon Health and Science University, Portland, OR 97201 USA; 2 Knight Cancer Institute, Oregon Health and Science University, Portland, OR 97201 USA; 3 Division of Oncology, Department of Medicine, Stanford University School of Medicine, Stanford, CA 94305 USA; 4 Department of Molecular & Cellular Endocrinology, Diabetes and Metabolism Research Institute, City of Hope Medical Center, Duarte, CA 91010 USA; 5 Collaborative Center for Translational Mass Spectrometry, Translational Genomics Research Institute, 445 N 5th St, Phoenix, AZ 85004 USA; 6 Brenden-Colson Center for Pancreatic Care, Oregon Health and Science University, Portland, OR 97201 USA; 7 Molecular & Cellular Biology, Oregon State University, Corvallis, Oregon 97331 USA; 8 Comprehensive Cancer Center, Beckman Research Institute, City of Hope Medical Center, Duarte, CA 91010 USA; 9 Department of Systems Biology, Beckman Research Institute of the City of Hope, Monrovia, CA 91016 USA; 10 Department of Hematological Malignancies, Beckman Research Institute of City of Hope, Duarte, CA 91010 USA

**Keywords:** c-MYC, BCR-ABL, RAS, Lymphoma, T-ALL, Cancer metabolism, Lipogenesis, Fatty acid synthesis, Oncogene addiction, ACACA, FASN, De novo lipogenesis

## Abstract

**Background:**

Metabolic reprogramming is a central feature in many cancer subtypes and a hallmark of cancer. Many therapeutic strategies attempt to exploit this feature, often having unintended side effects on normal metabolic programs and limited efficacy due to integrative nature of metabolic substrate sourcing. Although the initiating oncogenic lesion may vary, tumor cells in lymphoid malignancies often share similar environments and potentially similar metabolic profiles. We examined cells from mouse models of MYC-, RAS-, and BCR-ABL-driven lymphoid malignancies and find a convergence on *de novo* lipogenesis. We explore the potential role of MYC in mediating lipogenesis by ^13^C glucose tracing and untargeted metabolic profiling. Inhibition of lipogenesis leads to cell death both *in vitro* and *in vivo* and does not induce cell death of normal splenocytes.

**Methods:**

We analyzed RNA-seq data sets for common metabolic convergence in lymphoma and leukemia. Using *in vitro* cell lines derived in from conditional MYC, RAS, and BCR-ABL transgenic murine models and oncogene-driven human cell lines, we determined gene regulation, metabolic profiles, and sensitivity to inhibition of lipogenesis in lymphoid malignancies. We utilize preclinical murine models and transgenic primary model of T-ALL to determine the effect of lipogenesis blockade across BCR-ABL-, RAS-, and c-MYC-driven lymphoid malignancies. Statistical significance was calculated using unpaired *t*-tests and one-way ANOVA.

**Results:**

This study illustrates that *de novo* lipid biogenesis is a shared feature of several lymphoma subtypes. Using cell lines derived from conditional MYC, RAS, and BCR-ABL transgenic murine models, we demonstrate shared responses to inhibition of lipogenesis by the acetyl-coA carboxylase inhibitor 5-(tetradecloxy)-2-furic acid (TOFA), and other lipogenesis inhibitors. We performed metabolic tracing studies to confirm the influence of c-MYC and TOFA on lipogenesis. We identify specific cell death responses to TOFA *in vitro* and *in vivo* and demonstrate delayed engraftment and progression *in vivo* in transplanted lymphoma cell lines. We also observe delayed progression of T-ALL in a primary transgenic mouse model upon TOFA administration. In a panel of human cell lines, we demonstrate sensitivity to TOFA treatment as a metabolic liability due to the general convergence on *de novo* lipogenesis in lymphoid malignancies driven by MYC, RAS, or BCR-ABL. Importantly, cell death was not significantly observed in non-malignant cells *in vivo*.

**Conclusions:**

These studies suggest that *de novo* lipogenesis may be a common survival strategy for many lymphoid malignancies and may be a clinically exploitable metabolic liability.

**Trial registration:**

This study does not include any clinical interventions on human subjects.

**Supplementary Information:**

The online version contains supplementary material available at 10.1186/s40170-021-00263-8.

## Introduction

Cancer cells can be defined by their constant and uncontrolled proliferation. This requires increased need for cellular building blocks such as amino acid synthesis, nucleotide production, and *de novo* lipid synthesis [1, 2]. To achieve this, cancer cells must reprogram their metabolic pathways to accumulate intermediates as sources for these building blocks. Thus, altered metabolism is a hallmark of cancer [3]. For example, glucose metabolism is commonly altered to decouple glycolysis from pyruvate oxidation (Warburg effect) [4, 5]. Although glycolysis is much less energy efficient than aerobic respiration, yielding 2 ATP instead 32 ATP, it provides a surplus of metabolic substrates that aerobic respiration does not, thereby providing a cellular growth advantage to cancer cells [6, 7]. Additionally, lactate production from glycolysis has been shown to alter the intracellular redox balance thereby promoting invasiveness [5, 8, 9].

Efforts have been made to exploit these metabolic dependencies ever since the Warburg effect was first described [5, 10, 11]. Despite the identification of several therapeutic targets, many glycolysis inhibitors show toxicity in normal tissues and/or limited therapeutic response [10]. In cancer cells, lipid metabolism may be altered where intermediate substrates from glycolysis are diverted from energy production to biosynthesis of fatty acids [5, 12, 13]. These fatty acids are utilized to create membranes and signaling molecules such as phospholipids, sterols, and other lipids [5, 14, 15]. All of these *de novo* lipid production pathways begin with the rate-limiting conversion of acetyl-CoA to malonyl-CoA, facilitated by acetyl-CoA carboxylase (ACC or ACACA) [16, 17], and the concomitant conversion to palmitate by fatty acid synthase (FASN), representing the committed step in *de novo* lipid biogenesis. In this study, we utilize cell lines derived from conditional murine models that drive several cancer subtypes [18–22]. These models contain a Tet-O system where the addition of doxycycline halts expression of RAS, c-MYC (hereafter MYC), or BCR-ABL, permitting perturbation studies of the driving oncogene [22–24]. We employ cell lines derived from these models to explore common metabolic features in the context of MYC, RAS, and BCR-ABL-driven lymphoid malignancies. Although cancer cells have previously been shown to be sensitive to lipogenesis inhibitors, oncogene driver-based susceptibility has not been examined closely [25]. In this study, we examine the effects of ACACA inhibition in lymphomas using 5-(tetradecloxy)-2-furic acid (TOFA), and other inhibitors of the fatty acid (FA) synthesis pathway, resulting in shutdown of lipid biogenesis [17, 26, 27].

Despite being driven by different oncogenes, we detect increased lipid biogenesis profiles in all three models. These results are recapitulated in malignant human lymphoid cell lines. We find that each driver oncogene is sensitive to blockade of lipogenesis in both mouse and human lymphoid malignancies. Importantly, cell death programs initiated by lipogenesis blockade are specific to malignant cells *in vivo*. In summary, we describe a potential metabolic liability in lymphoid malignant cells due to their convergence and reliance on *de novo* lipid biogenesis.

## Results

### MYC, BCR-ABL, and RAS regulate lipogenesis

**Fig. 1:**
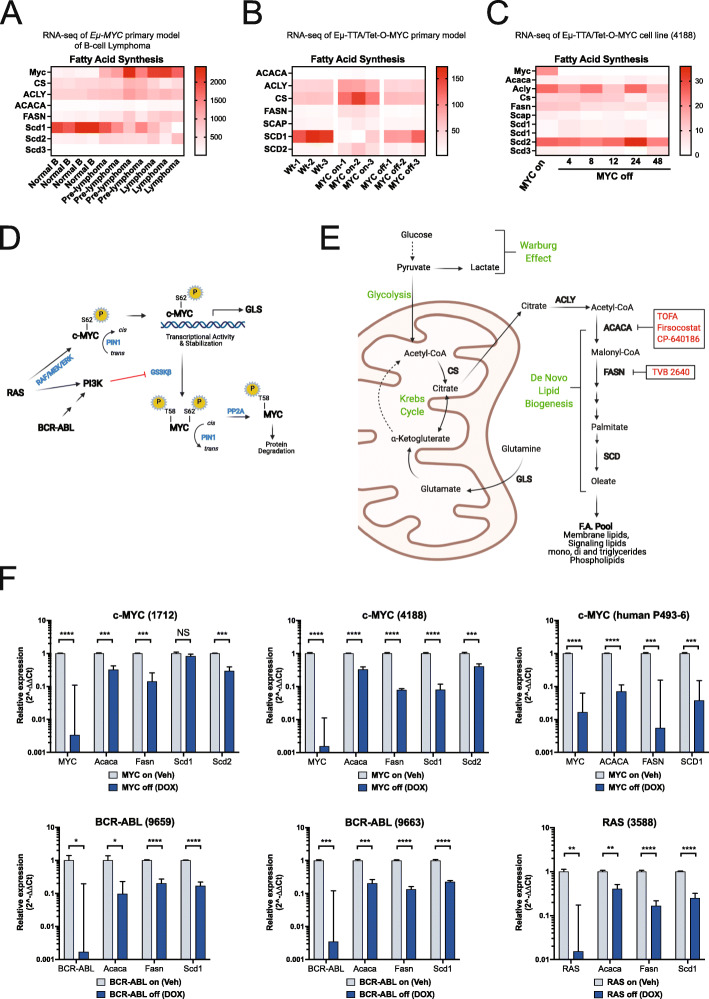
MYC regulates lipogenesis genes. **A** Eμ-MYC model of B cell lymphoma RNA-seq reveal upregulation of de novo fatty acid lipogenesis genes linked to MYC expression as the B cell lymphoma progresses (GSE 51011). **B** RNA-seq profiles of lipogenesis genes from *Eμ-tTA/Tet-O-MYC* model T-ALL. Data mining experiments of MYC on vs off state in the *Eμ-tTA/Tet-O-MYC* primary model of T-ALL, inclusive of the FVB/N background shows MYC-dependent regulation of lipogenesis genes in vivo*.* (GSE106078). **C** RNA-seq was performed on cell lines (4188) derived from *Eμ-tTA/Tet-O-MYC* transgenic model in a MYC-off time-course. Decreased expression values were observed for fatty acid synthesis pathway genes (GSE178580) after MYC expression is abrogated. **D** Oncogenic MYC is stabilized by S62 phosphorylation and PIN1 mediated isomerization (for review see Cohn et al. [30]). GSK3beta initiates MYC recycling and is inhibited by both RAS and BCR-ABL, leading to a potential convergence on MYC-mediated programs. MYC is known to activate transcription of GLS which facilitates the entry of glutamine into the Krebs cycle. **E** Graphical representation of the fatty acid synthesis pathway, and chemical inhibitors of the de novo fatty acid synthesis are shown (red). **F** Cells derived from conditional transgenic murine models with differing oncogenic drivers. Conditional expression of the initiating oncogene is controlled by a Tet-Off system. The suppression of MYC or BCR-ABL or RAS results in reduced mRNA expression of specific genes known to facilitate de novo lipogenesis pathway, indicating a common feature of lymphoid malignancies regardless of the initiating oncogene. Values were normalized to ubiquitin controls and reported as fold change relative to MYC on condition (vehicle). *P⩽0.1; **P ⩽ 0.01; **P ⩽ 0.001; ***P ⩽ 0.001; ****P ⩽ 0.0001

We began by examining lipogenesis gene regulation in two transgenic conditional MYC expressing murine models. In an *Eμ-Myc* model [28, 29] that drives B cell lymphoma, we profiled several pathways important to lipid biosynthesis via RNA-seq data mining experiments (supplementary Fig. 1A). We observed FA synthesis genes were particularly upregulated during lymphoma progression driven by MYC (Fig. 1A). RNA-seq data [31] using a conditional *Eμ-tTA/Tet-O-MYC* murine model of T-ALL [22] show similar results (Fig. 1B) with the notable exception of Stearoyl-CoA desaturase (Scd1), although the Scd2 paralog does follow MYC expression patterns.

Using a cell line established from the *Eμ-tTA/Tet-O-MYC*-conditional transgenic murine model (4188 line) [22], we explored genome-wide transcriptional changes when MYC expression is revoked over several timepoints. We profiled multiple pathways involved in lipid synthesis (supplementary Fig. 1B) and observed that *de novo* fatty acid synthesis genes exhibited reduced expression patterns correlating with abrogated MYC expression over time (Fig. 1C).

MYC is a central mediator of many oncogenic programs and known to be activated and stabilized by RAS and stabilized by BCR-ABL (Fig. 1D) [32–36]. A more complete review of oncogenic MYC stabilization is discussed elsewhere [30]. We examined publicly available microarray dataset of human BCR-ABL-driven cell lines treated with the ABL inhibitor Imatinib (GSE 23743) [37]. Inhibition of BCR-ABL with Imatinib predominately reduced lipogenesis gene expression (supplementary Fig. 1C), although the Nalm1 cells show increased ACACA and FASN expression. Three of four cell lines also show reduced MYC expression when treated with Imatinib, with the exception of the BV173 which displayed high MYC and lipogenesis pathway expression. This suggests that MYC expression levels may influence lipogenesis genes even in BCR-ABL-driven cells.

ChIP-seq data from conditional MYC expressing human Burkitt’s lymphoma line P493-6 [38] reveal direct MYC binding at promoter regions of lipogenesis genes (supplementary Fig. 2A). ChIP-seq data in U2OS cells expressing conditional MYC [39] show direct binding of MYC to ACC, but not to other FA genes, implying that MYC occupancy at FA genes is coordinated by tissue subtype (supplementary Fig. 2B) and is not a general feature of MYC biology.

Recent studies in other cancers have shown reliance on the lipogenesis pathway [40–46]. In order to determine if reliance on lipogenesis is a general feature in lymphomas, we examined other oncogene-driven lymphoid malignant models such as BCR-ABL and RAS [20, 21, 24]. We obtained cell lines derived from conditional Tet-O transgenic murine models of lymphoma driven either by MYC, RAS or BCR-ABL [20–22, 24] and examined early and late FA synthesis gene profiles. Acaca converts acetyl-Co-A into malonyl-Co-A, followed by Fasn conversion of either acetyl-Co-A or malonyl-Co-A into palmitate synthesis and represents the committed step in *de novo* fatty acid synthesis (Fig. 1E). Further downstream Scd converts long chain saturated fatty acid into oleic acid (oleate). The mouse genome encodes 3 different Scd enzymes (SCD1,2,3) and the human genome encodes a single SCD enzyme. We examined two cell lines derived from the primary *Eμ-tTA/Tet-O-MYC* model (4188 and 1712), the human B cell line P493-6, two cell lines (9659 and 9663) derived from the *Eμ-tTA/Tet-O-BCR-ABL* model, and one cell line (3588) derived from the *Eμ-tTA/Tet-O-RAS* model to perturb oncogene expression and explore potential influence on lipid biogenesis gene expression.

Examination of the MYC ON versus OFF state reveals MYC positively regulates expression of Acaca, Fasn, and Scd2 in both 4188 and 1712 cell lines (Fig. 1F). The human P493-6 MYC cell line displays a similar pattern. Interestingly, we found comparable metabolic regulation in RAS and BCR-ABL-driven cell lines, suggesting a potential common metabolic convergence in lymphoma and T-ALL (Fig. 1F). To address the potential influence on FA synthesis by doxycycline (dox), we transduced the murine 4188 cell line and the human P493-6 cell line with MYC overexpression vectors, and a mutant MYC-V394D substitution that disrupts interaction with Miz1, a transcriptional co-regulator of MYC-dependent programs [47, 48]. MYC overexpressing cells treated with dox exhibited a reduction in MYC expression returning to the empty vector control levels (supplementary Fig. 3A, B), presumably due to modulation of the parental transgene. We observed either non-significant or greatly reduced effect on downregulation of the lipogenesis genes in response to dox treatment, compared to the parental lines (Fig. 1F). Interestingly, the MYC-VD mutant showed significant effect for FASN in both the 4188 and P493-6 cells, suggesting a direct regulation of FASN by MYC, consistent with the ChIP-seq tracks (supplementary Fig. 2)

Taken together, these results suggest that MYC, RAS, and BCR-ABL-driven lymphoma cells upregulate lipid biogenesis pathways, which may be a common feature across lymphoid malignancies. Importantly, MYC has been implicated as a mediator of both RAS and BCR-ABL-driven cancers (Fig. 1D) [26–29, 31] and is shown to directly influence gene expression of ACACA, FASN, and SCD.

### Inhibition of lipogenesis by TOFA suppresses cell proliferation

**Fig. 2:**
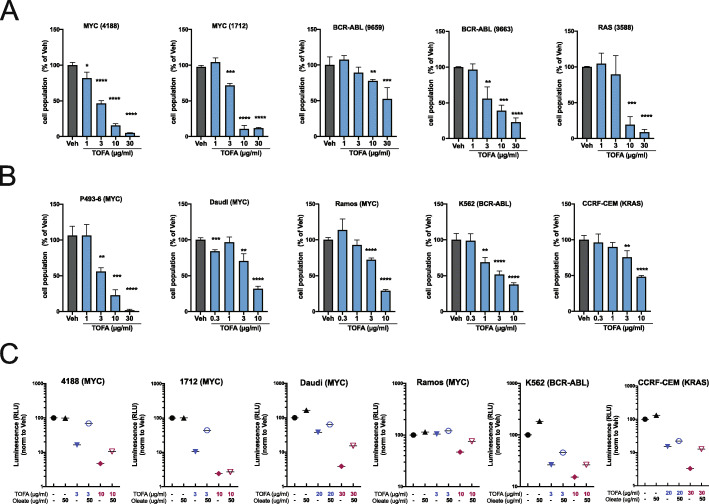
Inhibition of lipogenesis suppresses cell growth in lymphoid malignancies. **A** Cells derived from conditional transgenic murine models with differing oncogenic drivers. Cell populations in oncogene-dependent lymphoid malignancies are sensitive to lipogenesis inhibition with TOFA, with MYC cells displaying the greatest cell population decline. **B** Human cell lines with differing oncogenic drivers are sensitive to lipogenesis inhibition. Values were normalized to background controls and reported as percent cell population relative to vehicle control. *P ⩽ 0.1; **P ⩽ 0.01; **P ⩽ 0.001; ***P ⩽ 0.001; ****P ⩽ 0.0001. **C** Oleate rescue experiments. Palmitate long chain saturated fatty acids are converted into oleic acid (oleate) by SCD. Cells pre-treated with oleate (90 min) and dosed with the lipogenesis inhibitor TOFA are partially or fully rescued in all cells tested, suggesting the inhibitor is effecting de novo fatty acid synthesis. Error bars are indicated by vertical lines from sample reads across 4 technical replicates

To ascertain whether MYC, RAS, or BCR-ABL-driven cell lines are sensitive to blockade of the lipogenesis pathway, we used the Acaca inhibitor TOFA [17, 26]. TOFA blocks the synthesis of malonyl-CoA thereby inhibiting fatty acid synthesis pathway and suppresses tumor growth in prostate, ovarian, lung, and colon cancer cell lines [49–51]. Side effects of TOFA are predominately altered metabolism due to FA synthesis inhibition [27]. We dosed the conditional cell lines with TOFA, which reduced viable cell populations (Fig. 2A) in all three oncogene-driven cells.

We also examined several human cell lines. The P493-6 lymphoma cells also show dependency upon lipid biogenesis with TOFA resulting in reduced cell numbers (Fig. 2B). We then tested malignant lymphoid lines of human origin driven by MYC (Daudi, Ramos), KRAS^G12D^ (CCRF-CEM), and BCR-ABL (K562). All cell lines displayed sensitivity to lipogenesis inhibition by TOFA (Fig. 2B). To verify pathway specificity of TOFA, rescue experiments with oleate pre-treatment before dosing with TOFA were conducted. A partial or complete rescue was achieved in all cell lines tested, indicating FA pathway-specific response to TOFA (Fig. 2C).

We then examined other clinical inhibitors of ACACA (firsocostat and CP-640186) or FASN (TVB-2640) (see Fig. 1E) [27]. In clinical trials, Firsocostat is effective in treating nonalcoholic steatohepatitis [52–54]. CP-640185, a nonselective ACACA inhibitor [55–57], equally inhibits rat, monkey, mouse, and human ACACA. We performed dose-response experiments in Daudi, Ramos, CCRF-CEM, and K562 cells with each of the lipogenesis inhibitors (supplementary Fig. 4A). Both TOFA (ACACA inhibitor) and TVB-2460 (FASN inhibitor) [58–60] had similar profiles for sensitivity, with CP-640186 to a slightly lesser extent. Firsocostat had mild effect on cell growth at only the highest doses, and in our hands did not appear to be a potent inhibitor of lipogenesis blockade. We also attempted to utilize Cas9-mediated genomic disruption of ACACA and FASN expression in the Daudi, Ramos, CCRF-CEM, and K562 cell lines using GeCKO library sgRNA guides combined as a pool. However, the cell lines struggled, and we were only able to obtain a significant reduction in FASN (63% of EMV control) in the Ramos cell lines (supplementary Fig. 4B). As expected, in these cells, we observed attenuated sensitivity to TOFA treatment.

TOFA inhibits cell growth in prostate, ovarian, lung, and colon cancer cell lines [49–51], but is not pan-cancer specific [27]. Three colon cancer cell lines were tested for TOFA sensitivity (DLD1^MYC^, HCT116^CDKN2B^, RKO^BRAF^) and were moderately sensitive to prolonged TOFA treatment at high concentrations (supplemental Fig.5A). None of the cell lines achieved an ED50 within the 48 h timepoint as compared to lymphoid cells (supplementary Fig.4A). Three KRAS-dependent pancreatic cancer cell lines (HPAFII, MIA PaCa-2, PANC-1) were subjected to TOFA treatment. Both HPAFII and MIA PaCa-2 cell lines achieved ED50 between 48 and 54 h, while the PANC1 cell line displayed moderate sensitivity to TOFA treatment (supplementary Fig.5B). The hepatoblastoma cell line HepG2 (chromosomal amplified) are largely resistant to TOFA treatment (supplementary Fig. 5C).

While lipogenesis inhibition may be an effective strategy in many cancer subtypes, this approach is likely context dependent, as variable responses were seen in this panel of cancer cells. In this study, we see broad sensitivity to lipogenesis inhibition in RAS, MYC, and BCR-ABL-driven lymphoid malignancies, suggesting general convergence and a potential metabolic liability in lymphomas and leukemias.

### Loss of MYC results in reduced lipid biogenesis.

**Fig. 3:**
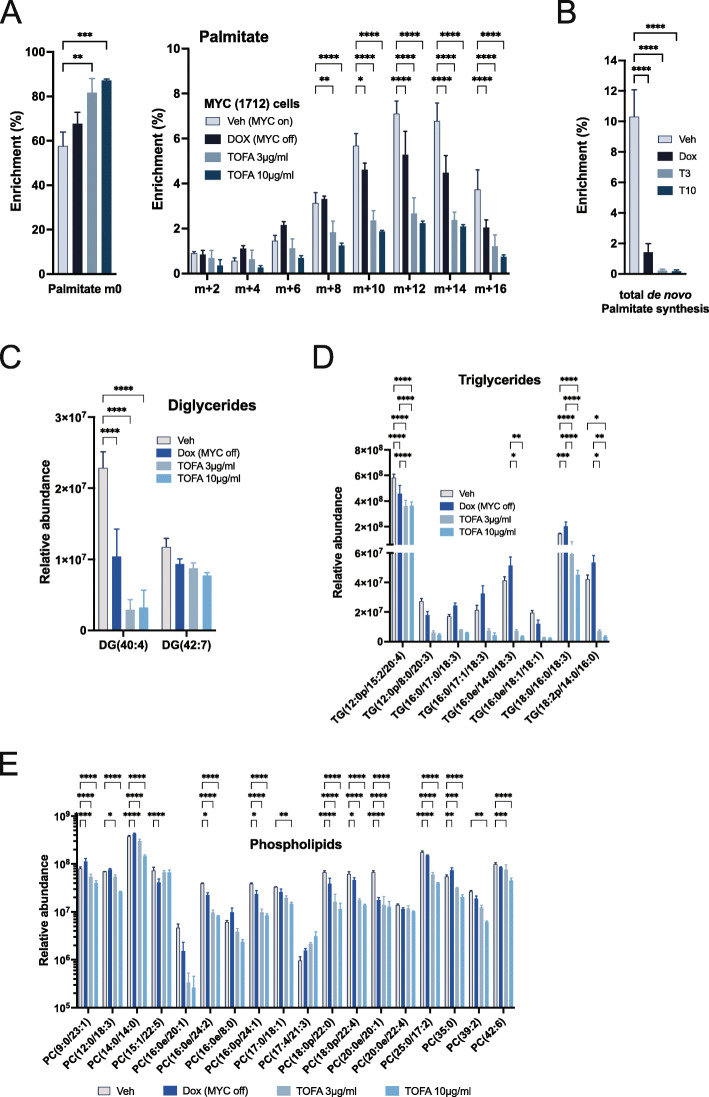
MYC expression enhances de novo Fatty Acid synthesis. **A**-**B**) Carbon tracing experiments (U-13C) were conducted with labeling under normal culturing conditions. Cells were either treated with doxycycline or vehicle for 8 hours, followed by treatment with either dox, TOFA 3μg/ml or 10μg/ml for 24hrs. Palmitate tracing was conducted as previously described (62). **A**) Glucose tracing shows that de novo fatty acid synthesis is significantly lower in dox-treated cells, which is further repressed by TOFA treatment. Statistical analysis for m0 used ordinary onewayANOVA, and m+2 through m+16 utilized 2-way ANOVA. **B**) Total de novo palmitate synthesis levels from labeled Glucose. **C**-**E**) Untargeted Lipidomics: Similar experiments on 1712 MYC-dependent cells were subjected to an untargeted lipidomics profiling. **C**) Upon dox or TOFA (3μg/ml or 10μg/ml) for 24hrs, longer chain DGs are significantly lower compared to vehicle. **D**-**E**) Majority of the medium chain TGs and PCs also followed similar trends as observed in DGs which may have resulted from altered status of FASN, ACC and SCD. Statistical significance was determined by 2-way ANOVA

As we observed quite striking effects on gene expression profiles for key *de novo* lipid biogenesis genes, we sought to confirm the actual metabolic profiles using the murine 1712 cell line allowing modulation of MYC expression. We performed ^13^C tracing [61] for MYC ON (veh) versus MYC OFF, and two concentrations of TOFA and profiled for palmitate chain elongation. [U-^13^C] glucose tracing shows that *de novo* fatty acid synthesis is significantly lower in dox-treated cells, which is also potently repressed by TOFA treatment (Fig. 3A). Strikingly, the total palmitic acid derived *de novo* from glucose is significantly abrogated in MYC OFF- or TOFA-treated cells (Fig. 3B).

To examine lipid biogenesis more broadly, we performed untargeted lipidomics [62] on the 1712 MYC-driven cell line. We identified 3171 lipid ions corresponding to 1629 lipid species belonging to several lipid classes including mono-, di-, and triglycerides (MG, DG, and TG), phospholipids (PCs, PE, LysoPCs), cardiolipins, hydroxyl fatty acids, sphingolipids, and ceramides. We observed significant alterations in each lipid class for dox, TOFA 3 μg/ml, and TOFA 10 μg/ml groups compared to vehicle. For this analysis, we focused on DG, TG, and PCs to determine the effect of MYC OFF on lipogenesis as a result of altered expression of upstream players of lipid biosynthesis pathways, i.e., FASN, ACACA, and SCD. In the MYC OFF, TOFA 3 μg/ml, TOFA 10 μg/ml treatment, and longer chain DGs (Fig. 3C) are significantly lower compared to vehicle. Most longer chain PCs (C37-C42) and three out of eight TG species (TG(47:6), TG(40:3), and TG(C52:2)) followed the same trend as DGs (Fig. 3D). However, some PCs (C24-C33) and TGs were increased in MYC OFF (Fig. 3E), suggesting that MYC may regulate the biosynthesis of PCs and TGs based on the length of their acyl chains [63, 64]. Alternatively, the increase in TGs and PCs may be a result of altered catabolic phospholipase activity or acyl chain elongation in response to altered energy demand when MYC is revoked [65]. TOFA seemed to have a higher impact on lowering DGs, TGs, and PCs compared to MYC inhibition. In summary, we performed ^13^C tracing experiments and untargeted lipidomics to determine if the regulation of lipogenesis genes by MYC is comparable to direct inhibition of lipogenesis and show compelling evidence of this relationship.

### Inhibition of lipogenesis leads to apoptosis

**Fig. 4:**
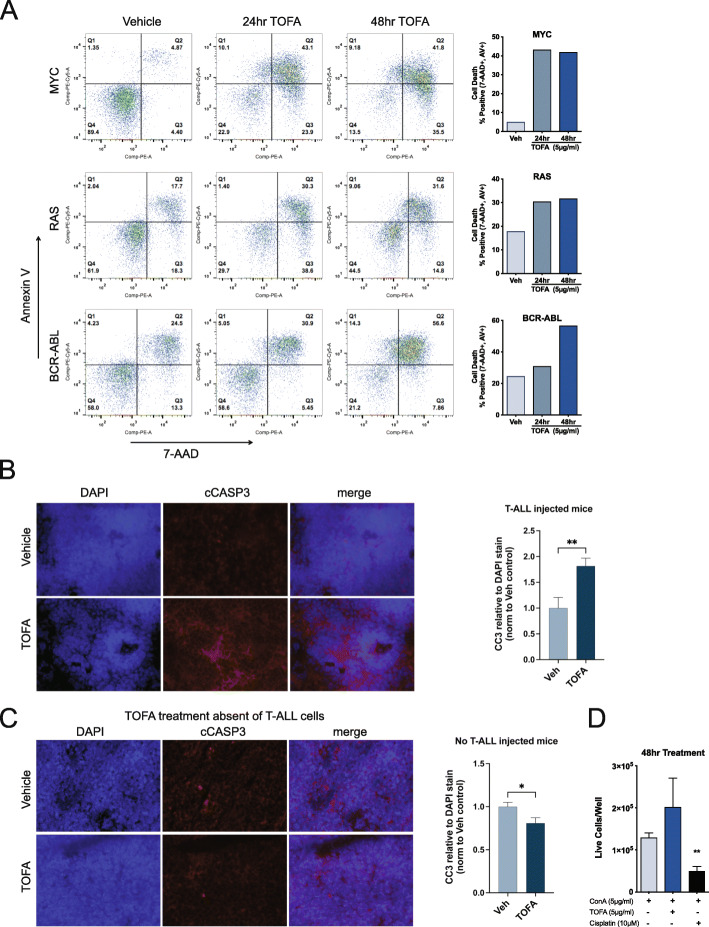
Inhibition of lipogenesis results in cell death. **A** TOFA treatment in either MYC, RAS, or BCR-ABL-dependent lymphoid results in significant increased cell death as detected by Annexin V and 7-AAD co-staining (quadrant 2) over t24 and 48 h timepoints (TOFA 5.5 μg/ml). Graphical representation of data to the right of flow plots. **B** NSG mice were intravenously injected with MYC-driven T-ALL cells derived from *Eμ-tTA/Tet-O-MYC* transgenic model and treated with TOFA for 4 days on, followed by 4 days off and then sacrificed. Cleaved caspase 3 is significantly more prevalent in splenic tissue from TOFA-treated mice when compared to control. **C** Spleens from mice that did not receive T-ALL cell injections but were treated with TOFA showed slightly less CC3 signal compared to vehicle control, suggesting that TOFA is only induced apoptosis in T-ALL cells in vivo. **D** Spleens were harvested from the wild-type FVB/N mice (transgenic background), RBC were lysed, and splenocytes were activated with ConA, followed by indicated treatments. TOFA treatment did not significantly impair the activated splenocyte population compare to the ConA alone control. Comparison to ConA control utilized an unpaired *t*-test **P ⩽ 0.01

To further characterize the observed reductions in cell populations upon TOFA treatment, we measured cell death by flow cytometry. Co-stains for 7-AAD and Annexin V revealed increased apoptosis in MYC (8.6 fold), RAS (1.7 fold), and BCR-ABL (2.3 fold) cells (average of two timepoints) treated with TOFA (Fig. 4A, extended data in supplementary Fig 6A-C). We examined apoptosis by immunofluorescence (IF) in spleens from NOD-SCID IL-2Rγ^−/−^ (NSG) mice implanted IV with 4188 (MYC) and treated with TOFA for 4 days. We detected 1.8-fold increase for the apoptosis marker cleaved caspase-3 (CC3) in TOFA-treated animals compared to vehicle control (Fig. 4B). Co-staining with the T cell marker CD4 confirmed that T-ALL cells (CD4 expressing) were correlated with CC3-positive cells (supplementary Fig. 7A). We also examined the CC3 background staining observed in vehicle-treated mice show the signal is not due to autofluorescence (supplementary Fig. 7B). Importantly, mice that were not recipients of exogenous cells (4188) and treated with TOFA displayed slightly less CC3 staining than vehicle control (Fig. 4C), indicating that apoptosis induced by FA inhibition primarily targets the transplanted lymphoma cells, in this case MYC-driven T-ALL.

To further characterize the observation that TOFA specifically targets metabolically reprogramed cancer cells in our models, we examined normal proliferating splenocytes. Spleens from the wild-type background FVB/N mice were harvested and splenocytes activated with concanavalin A (ConA). TOFA treatment did not cause a significant reduction in proliferation or cell density when compared to cisplatin (Fig. 4D). These data provide compelling evidence that MYC-driven T-ALL cells are sensitive to lipogenesis pathways for survival both *in vitro* and *in vivo*, and cell death in lymphocytes is not a general consequence of TOFA treatment.

### Evaluation of unfolded protein response stress

Several studies have linked changes in lipid metabolism to endoplasmic reticulum stress (ER stress) and unfolded protein response stress (UPR stress) [66–71]. We examined genes commonly upregulated in response to UPR stress in TOFA-treated conditional expressing murine cell lines (supplementary Fig. 8A). The C/EBP homologous protein (CHOP) transcript is activated by ER stress and was upregulated in each of the MYC-, RAS-, and BCR-ABL-driven cell lines, with the strongest response seen in the MYC-dependent cell lines. MYC-dependent cells displayed upregulation of activating transcription factor ATF6α, which activates UPR target genes [68]. The BCR-ABL-driven lines exhibited upregulation of HSP-α5, involved in degradation of misfolded proteins, and XBP1, which is activated by unfolded proteins in the ER. RAS-driven lymphoma cell lines actually downregulated XBP1, ATF6α, and HSP-α5. These data indicate that while UPR stress can be associated with inhibition of lipogenesis, we did not observe a shared response in the different oncogene-driven cells.

We also injected luciferase-labeled MYC-dependent cell lines into recipient NSG mice and treated the mice with either TOFA or vehicle. When the mice were moribund due to disease burden, we collected spleens from euthanized animals and examined UPR stress genes *in vivo* (supplementary Fig. 8B). We did not observe significant upregulation of UPR stress genes in TOFA-treated mice compared to control. Interestingly, we also observed a reduction of the transgenic MYC, indicating a reduction of MYC-dependent cells in the presence of TOFA. However, our observations do not provide clear evidence of UPR stress involved in observed metabolic liabilities shown in Figs. 2 and 4.

### Inhibition of lipogenesis delays engraftment, progression, and splenic infiltration

**Fig. 5:**
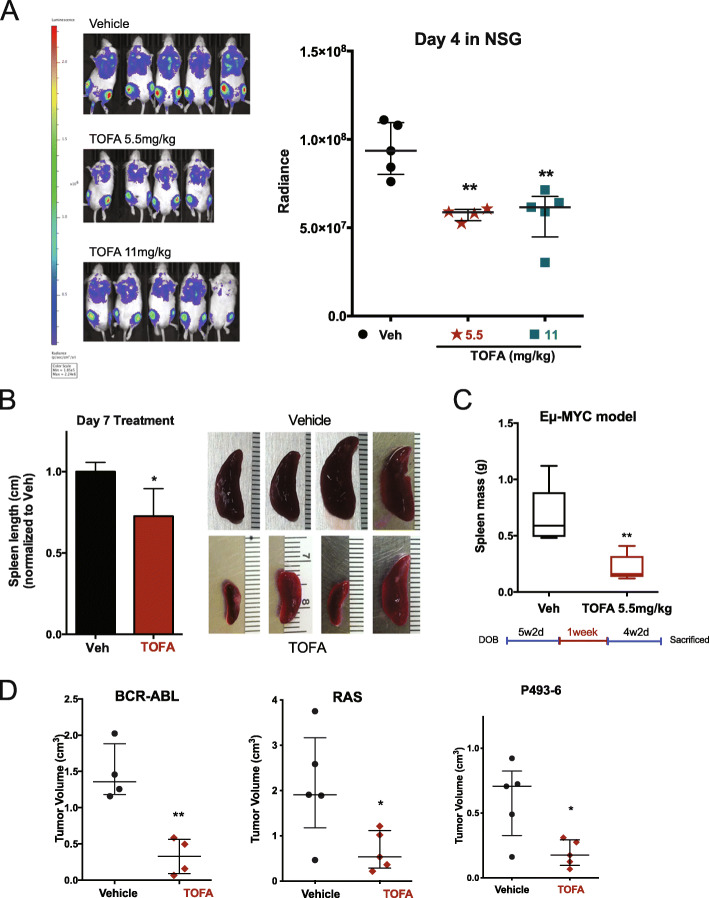
TOFA treatment delays engraftment and splenic infiltration. **A** Luciferase-labeled MYC-driven lymphoma cells from *Eμ-tTA/Tet-O-MYC-*conditional transgenic murine model were intravenously injected into NSG mice and quantitated over time using bioluminescence imaging (BLI). Mice treated with TOFA show significantly decreased signal indicating reduced engraftment. **B** Further analysis of the spleens from the mice utilized in panel **A** revealed significantly reduced spleen size in TOFA-treated animals compared to vehicle control, suggesting decreased infiltration. **C** *Eμ-tTA/Tet-O-MYC* (primary model of T-ALL) mice were treated with TOFA at 5 weeks of age for 1 week and tracked for an additional 4 weeks. TOFA-treated mice were less moribund and exhibited significantly reduced spleen mass, suggesting inhibition of lipogenesis results in reduced disease onset and severity. **D** RAS and BCR-ABL driven T-ALL cell lines were allografted subcutaneously into NSG mice. We detected significantly reduced tumor volume for mice treated with TOFA. We also observed attenuated tumor volume using the human B cell lymphoma cell lines P493-6 subcutaneously xenografted on NSG mice. Comparisons to vehicle control utilized unpaired *t*-test. *P ⩽ 0.1; **P ⩽ 0.01

We next investigated possibility that inhibition of lipogenesis may delay engraftment and cancer progression *in vivo*. We implanted MYC-driven luciferase-labeled 4188 cells in recipient mice and observed a reduction in tumor burden for TOFA-treated mice, measured by bioluminescence imaging (BLI) (Fig. 5A), confirming that MYC-dependent cells are metabolically sensitive to lipogenesis inhibition *in vivo*. Spleen size at terminal endpoints was measured to determine effects on infiltration of T-ALL cells (Fig. 5B). TOFA-treated mice exhibited a reduced spleen size compared to vehicle-treated mice, indicating reduced T-ALL disease burden.

In the primary *Eμ-tTA/Tet-O-MYC* transgenic mouse model, mice were treated at 5 weeks of age just prior to visible onset of disease with either TOFA or vehicle for 1 week. The mice were monitored for 4 weeks with no further treatments and sacrificed when control mice developed T-ALL as determined by hunched posture, enlarged lymph nodes, and ruffled fur. To evaluate disease progression, we measured splenic infiltration of T-ALL cells by bulk spleen mass. At 4 weeks post injection with TOFA, delayed progression of the primary transgenic model of MYC-dependent T-ALL was exhibited (Fig. 5C). We also subcutaneously injected BCR-ABL (9663), RAS (3588), and the MYC-dependent B cell lymphoma cell line P493-6 into NSG mice. In all three oncogene-dependent cell lines, TOFA treatment (IP) reduced tumor progression (Fig. 5D). These results suggest a common metabolic vulnerability in malignant lymphoid cells that can be exploited *in vivo* by lipogenesis inhibitors such as TOFA.

### Inhibition of lipogenesis in human cell lines derived from hematopoietic malignancies

**Fig. 6:**
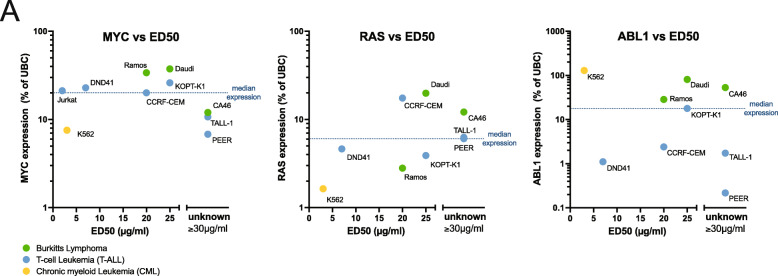
Oncogene expression and sensitivity to TOFA treatment in a limited panel of lymphoid cancer cell lines. **A** We obtained a panel of human lymphoma cells and calculated ED50 values (Supplemental Fig. 9A) and compared to measured relative MYC, RAS, and ABL1 expression levels (supplemental Fig. 10A). Median expression is indicated by dotted line. Cells resistant to TOFA treatment are indicated as greater than 30 μg/ml. MYC expression is associated TOFA sensitivity

Previously, we demonstrated that MYC-conditional Burkitt’s lymphoma cell line P493-6, and non-transgenic Daudi, Ramos, CCRF-CEM, and K562 human cell lines were sensitive to TOFA treatment (Fig. 2B). We expanded these studies on a limited panel of human cell lines derived from lymphoid malignancies (supplementary Fig. 9A). We found not all lymphoid malignant cells were sensitive to TOFA treatment. We then examined relative MYC, RAS, and ABL1 expression levels (Supplementary Fig. 10). For low or non-expressing cell lines, the error bars preclude any statistical grouping, a common limitation to qRT-PCR experiments. However, when we examine the TOFA-induced ED50 values (Supplementary Fig. 9A) with the median expression of each oncogene (Supplementary Fig. 10A), we observe a potential association with MYC expression in this specific panel of hematologic malignant cell lines (Fig. 6A). With the exception of the lone CML cell line tested (K562), expression patterns of MYC below the median of this limited panel were less sensitive to inhibition of lipogenesis.

## Discussion

We examined the convergence on *de novo* lipid synthesis in RAS, BCR-ABL, and MYC-driven hematopoietic tumor cells. We profiled RNA-seq and microarray data to discover which lipid synthesis pathways are modulated by MYC, and BCR-ABL, and found MYC-dependent regulation of *de novo* fatty acid biogenesis, even in some BCR-ABL-driven cells. Using 6 cell lines with conditional expression of either MYC, RAS, or BCR-ABL, we profiled the early committed steps in lipid biogenesis (Acaca and Fasn), and a terminal FA enzyme Scd. Both ACACA [46] and FASN [59] are of importance in preclinical models and are under examination in clinical trials [72–74].

We demonstrate that revoking oncogene expression results in downregulation of lipid biogenesis, similar to treatment with the Acaca inhibitor TOFA, and extend these studies to human lymphoid cell lines expressing mutant MYC, KRAS, or the BCR-ABL with similar results. We tested these human cell lines with multiple lipogenesis inhibitors and find that CP-640186 and TVB-2640 mirror the reduced cell populations observed in TOFA-treated cells (supplementary Fig. 4A). Rescue experiments with oleate- in TOFA-treated cells determined that this pathway is specific to *de novo* FA synthesis (Fig. 2C). ^13^C glucose tracing demonstrated that loss of MYC recapitulates inhibition of palmitate formation by TOFA treatment (Fig. 3A, B). Untargeted metabolic profiling revealed products of the FA synthesis such as lipid classes including mono-, di-, and triglycerides, phospholipids, cardiolipins, hydroxyl fatty acids, sphingolipids, and ceramides are all affected by loss of MYC, and TOFA treatment in the conditional MYC expressing 1712 cell line.

All three oncogene-driven models undergo apoptosis when treated with the FA inhibitor TOFA. We tracked tumor engraftment and progression via BLI and show that TOFA treatment reduced T-ALL disease burden (Fig. 5A), delayed tumor progression in established subcutaneous tumors in all three MYC-, RAS-, and BCR-ABL-driven cell lines, and substantially abrogate tumor progression in the primary model of MYC-driven T-ALL mice even after treatment regimens had halted (Fig. 5C). Strikingly, TOFA treatment did not result in increased apoptosis for NSG mice that were not implanted with MYC-driven tumor cells (Fig. 4D). Thus, our data suggests that TOFA is well-tolerated in normal splenocytes (Fig. 4C), and FA inhibition is specific to lymphoid malignant cells.

Of the three oncogene models tested, MYC is the only transcription factor, and we demonstrate MYC positively regulates ACACA, FASN, SCD, and metabolic intermediates. ChIP-seq data mining shows direct MYC binding to FA genes in Burkett’s lymphoma model cell line P493-6, but not in conditionally expressing MYC U2OS osteosarcoma cell lines (supplementary Fig.2), implying that sensitivity to FA inhibition is not a generalizable MYC hallmark, but rather may be a general program employed by lymphoid malignancies expressing high levels of MYC.

Although murine models are metabolically dissimilar to human metabolism, human cell lines recapitulate the sensitivity to FA inhibition by TOFA (Fig. 2B, supplementary 4A). MYC is stabilized by both BCR-ABL and RAS (Fig. 1D), and RAS also activates MYC through the Raf/Mek/Erk pathway leading to increased transcriptional activity [33, 35, 75, 76]. MYC has previously been described to regulate GLS which redirects glutamine pools into the Krebs cycle in cancer cells (Fig. 1E) [77]. Thus, MYC-mediated endpoints may play a central role in the convergence on lipogenesis.

We examined a limited panel of human hematologic malignant cell lines and profile TOFA sensitivity association with oncogene expression (Fig. 6A). These results together suggest that lymphoid cells with high MYC expression have exploited a metabolic strategy that relies upon fatty acid synthesis. Therefore, this metabolic convergence on *de novo* lipogenesis by MYC, RAS, and BCR-ABL tumors is potentially dependent on MYC stabilization and may provide rationale for development of more specific inhibitors and treatment strategies, especially considering the malignant cell-specific responses in these studies.

There are several unexplored areas to understand the mechanism of this metabolic liability in lymphoid malignancies. Although UPR induction was variable across the cell lines tested (supplementary Fig. 8), it is unknown whether or not specific components of UPR stress response contribute to the observed TOFA-induced apoptosis. As previously noted in literature, both BCR-ABL and KRAS can activate aspects of oncogenic c-MYC biology (Fig. 1D) [32–36], and it remains to be determined if MYC is central to lipogenesis inhibition sensitivity, or whether RAS and BCR-ABL activity are sufficient for metabolic reprograming. Further studies will be needed to elucidate the contribution of Miz1/MYC-dependent programs in regulating this pathway (supplementary Fig. 3A,B). Additionally, the role of oncogenic MYC in regulating apoptotic programs remains an enigma for the field at large and is likely to contribute to the mechanism of this metabolic liability in hematologic malignancies.

## Conclusion

These studies indicate that inhibition of lipogenesis may be a common survival strategy for many lymphomas and may be an exploitable metabolic liability in hematologic malignancies. Lipogenesis inhibition by TOFA is on-target, results in apoptosis, appears to be specific to lymphoid malignancies *in vivo* and is well-tolerated by normal splenocytes.

## Methods

### Cell culture conditions

Lymphoid cells lines were cultured in RPMI with L-glutamine (Gibco) supplemented with 10% fetal bovine serum (Tissue Culture Biologicals) with penicillin/streptomycin (Gibco) in a humidified 5% CO_2_ atmosphere. For mouse cell lines, 50 μM beta-mercaptoethanol was added to the media. Cells were maintained at 37 °C in a humidified incubator with 5% CO_2_, and typically passaged every 3 days. For HepG2 cells, DMEM (Gibco) was used in place of RPMI.

### RNA extraction and cDNA synthesis

RNA extraction from 2 × 10^7^ cells is done using the Qiagen RNEasy Extraction kit or a Zymo Research Quick-RNA Miniprep kits. RNA quality and concentration are assessed by a spectrophotometer, the Nanodrop. cDNA is then synthesized from 0.4 μg of the extracted RNA using Qiagen cDNA reverse transcription kit. The cDNA is then stored at − 20 °C.

### qPCR

Primers are designed by using NCBI PrimerBlast program and primer specificity was then verified using BLAST. Primers were generated by the Stanford PAN facility. Real-time PCR is performed in 96-well plates on an ABI Biosystems Thermo Cycler 7500. All primers are detected by using SYBR Green as fluorophore. Reactions are carried out in 20 μl that contained 1.5 μl cDNA, 0.5 μM forward and reverse primers, and 8 μL water and 10 μl of 2× SYBR Green master mix (ABI). Amplification cycle is as follows: 95 °C for 3 min, 35 cycles of 95 °C for 10 s, 63 °C for 30 s, 72 °C for 30 s, and a final extension at 72 °C for 5 min. At the end of the amplification cycles, a dissociation curve is done to verify non-specific amplification. The thermal cycler software generated threshold cycle (Ct) values for each gene; Ct is the number of cycles required to reach the threshold fluorescence 15 standard deviations above the noise. The Ct values are exported into Excel for analysis, and GraphPad Prism for statistical analysis.

**Table:** 

Primer table		
**Gene**		
MYC	Forward	CTGCGACGAGGAGGAGAACT
MYC	Reverse	GGCAGCAGCTCGAATTTCTT
Chop	Forward	CTGGAAGCCTGGTATGAGGAT
Chop	Reverse	CAGGGTCAAGAGTAGTGAAGGT
Xbp1 spliced	Forward	GACAGAGAGTCAAACTAACGTGG
Xbp1 spliced	Reverse	GTCCAGCAGGCAAGAAGGT
Atf6-alpha	Forward	AGCGCCCAAGACTCAAACC
Atf6-alpha	Reverse	CTGTATGCTGATAATCGACTGCT
Atf4	Forward	ATGGCGCTCTTCACGAAATC
Atf4	Reverse	ACTGGTCGAAGGGGTCATCAA
Eif2a	Forward	TACAAGAGACCTGGATACGGTG
Eif2a	Reverse	TGGGGTCAAACGCCTATTGATA
Scd1	Forward	TTCTTGCGATACACTCTGGTGC
Scd1	Reverse	CGGGATTGAATGTTCTTGTCGT
Fasn	Forward	GGAGGTGGTGATAGCCGGTAT
Fasn	Reverse	TGGGTAATCCATAGAGCCCAG
Ubc	Forward	AGCCCAGTGTTACCACCAAG
Ubc	Reverse	ACCCAAGAACAAGCACAAGG
Scd2	Forward	GCATTTGGGAGCCTTGTACG
Scd2	Reverse	AGCCGTGCCTTGTATGTTCTG
UBC	Forward	CTGGAAGATGGTCGTACCCTG
UBC	Reverse	GGTCTTGCCAGTGAGTGTCT
ABL1	Forward	TGAAAAGCTCCGGGTCTTAGG
ABL1	Reverse	TTGACTGGCGTGATGTAGTTG
FASN	Forward	AAGGACCTGTCTAGGTTTGATGC
FASN	Reverse	TGGCTTCATAGGTGACTTCCA
SCD	Forward	GCCCCTCTACTTGGAAGACGA
SCD	Reverse	AAGTGATCCCATACAGGGCTC
ACACA	Forward	TCACACCTGAAGACCTTAAAGCC
ACACA	Reverse	AGCCCACACTGCTTGTACTG
RAS	Forward	GAGTACAGTGCAATGAGGGAC
RAS	Reverse	CCTGAGCCTGTTTTGTGTCTAC

### RNA-seq

MYC-dependent cells derived from the primary *Eμ-tTA/Tet-O-MYC* murine model and maintained in culture. 100 × 10^6^ cells were seeded in T175 flasks in fresh media and timepoint samples began 24 h post seeding. Cells were dosed with 20 ng/ml doxycycline and samples collected at the indicated timepoints. RNA extraction included QIA shredder step, RNEasy kit, and on column DNA digestion (all reagents from Qiagen). Samples were shipped on dry ice to Beijing Genomics Institute (BGI) for the RNA-seq QC, sequencing pipeline, and read analysis.

### CellTiter-Glo assay

Cells were seeded at 10,000 cells per well in 96-well format in standard cell culture conditions described. Then, 24 and 48 h post seeding, cells were treated as indicated. At 72 h post seeding, cells were assayed using CellTiter-Glo assay (Promega G7570). After a 5 min incubation, cell populations were measured using spectramax (Molecular Devices). Background values were subtracted (media + CellTiter-Glo) and average of 4 wells were normalized to vehicle-treated cells. Statistical significance was determined using Student’s *t* test.

### CellTiter-Glo 2.0 assay

#### Drug treatment

Cell proliferation was obtained using the Cytation5 (BioTek) as a luminescence reader. Cells were plated at 3000 cells per well in 100 μL of media, and then let grow for 24 h in a 96-well white walled plate (Coring Incorporated Costar, 3610). After 24 h, cells were treated with TOFA (Cay10005263-1000, Cayman Chemical Company), Firsocostat (S8893, Selleck Chemicals), TBV-2640 (S9714, Selleck Chemicals), or CP-640186 (PZS0362, Millipore Sigma) at the following concentrations: .1, .3, 1, 3, 10, and 30 μg/ml. After either 24 or 48 h of growth under treatment, 50 μL of CellTiter-Glo 2.0 (G9242, Promega) is added to each well to be read. The plate was then rocked on a shaker for 2 min and then taken off the shaker and incubated at room temperature for 10 more minutes. The plate was then read on the Cytation5 (BioTek) at 6 luminescence fibers at 135 nm for 1 s per well.

#### Oleate rescue

Cell proliferation after a rescue experiment was obtained using the Cytation5 (BioTek) as a luminescence reader. Cells were plated at 10,000 cells/well at either 30 μg/ml, 50 μg/m of oleic acid (O3008, Sigma-Aldrich)-treated media, or plain media without the oleic acid. The oleic acid media was prepared by adding the oleic acid to the aliquots of media and then sonicating the solution for 60 s (duty 20, output 1). The proper number of cells were then spun down and resuspended with the either plain media or oleic acid media and plated in 96 white walled plates (Coring Incorporated Costar, 3610). Some wells were then treated 24 h later with either 20 μg/ml or 30 μg/ml of TOFA (Cay10005263-1000, Cayman Chemical Company). The plate was then read 24 h after TOFA treatment by adding 50 μL of CellTiter-Glo 2.0 (G9242, Promega) to each well to be read. The plate was then rocked on a shaker for 2 min and then taken off the shaker and incubated at room temperature for 10 more minutes. The plate was then read on the Cytation5 (BioTek) at 6 luminescence fibers at 135 nm for 1 s per well. Background values were subtracted (media + CellTiter-Glo) and average of 4 wells were normalized to vehicle-treated cells. Statistical significance was determined using Student’s *t* test.

### Annexin V and 7-AAD experiments

Cells were seeded in T25 flasks and treated as indicated. At 24 h post seeding/post initial treatment, remaining cells were treated to obtain 24 h timepoint. At 48 h post seeding/post initial treatment, cells were harvested for analysis. Briefly, 1 million cell aliquots from each treatment were collected and washed twice in cold PBS, followed by centrifugation at 1300 RPM. Cells were resuspended in 1× Annexin V Binding Buffer (BD Pharmingen). Cells were then stained with either no stain, 7-AAD (BD cat# 51-68981E), or PE Annexin V (BD cat# 5165875X) or 7-AAD and Annexin V for 1 h. Cells were then detected by flow cytometry (using BD FACS Aria SORP instrumentation). Data analysis was performed using FlowJo (TreeStar).

### Cleaved Caspase 3 immunostaining

O.C.T.-embedded tissue slides were prepared and stored at − 20. Slides were thawed at room temp (RT) for 30 min, and incubated at − 20 in acetone for 10 min, followed by air drying (10 min). 1× PBS rinse and Avidin block (Vector labs, Burlingame, Blocking kit cat #sp-2001) for 10 min were performed. Three minutes 3× PBS wash and 10 min Biotin block at RT were performed. 3× PBS wash followed by serum-free protein block (Dako cat # 2013-09) for 30 min at 37 °C was performed. Cleaved Caspase 3 antibodies (cell signaling #9661S) were diluted (1:100) in Dako antibody diluent (Cat #S202230-2CN) and incubated with tissue samples overnight at 4 °C. 3× PBS washes were followed by biotinylated anti-rabbit (Vector Labs Cat #BA-1000) 1:300D for 30 min at RT, 3× PBS washes and 1:300 cy3-streptavidin (Vector Labs cat #BMK-2202) for 30 min at RT, and 3× PBS washes and final wash with Vectashield (Vector Labs, Cat #H-1200) with DAPI followed by storage at 4 °C. Immunofluorescence signal was quantified by ImageJ (*n* = 3 representative images per condition), and CC3 signal normalized to corresponding DAPI channel, and statistical significance determined using Student’s *t* test.

### CD4 and CC3 IF co-stain

Immunofluorescent detection of cleaved caspase-3 (CC3) and CD4 antigen was performed on 10-μm-thick cryo-sections mounted on glass slides (Superfrost Plus, Fischer Scientific, Pittsburgh, PA). Post-thawing at room temperature for 30 min, the tissue was fixed in cold acetone for 10 min and endogenous biotin was blocked (Avidin/Biotin Blocking Kit, Vector Laboratories, Burlingame, CA). Tissue was further blocked in a protein serum (Dako, Carpinteria, CA) for 30 min and CC3 primary antibody (1:100, Cell Signaling, Danvers, MA #9661S) was applied overnight at 4 °C. Next day, sections were washed in PBS and incubated with biotinylated rabbit secondary antibody (1:300, Vector Laboratories, Burlingame, CA) for 30 min followed by incubation with CY-3 Streptavidin (1:300, M.O.M kit, Vector Laboratories, Burlingame, CA) for 30 min. The following day, tissues were re-blocked in protein serum for 30 min and incubated overnight at 4 °C with CD4 primary antibody (1:100, Santa Cruz, Dallas, TX # sc-13573). Next day, tissues were washed in PBS and incubated with Alexa Fluor 488 Goat anti-rat secondary (1:500, Life Technologies, Waltham, MA). The tissues were counterstained with Vectastatin DAPI mounting media (Vector Laboratories, Burlingame, CA).

### *In vivo* mouse experiments

Cell lines generated from *Eμ-tTA/Tet-O-MYC* transgenic murine model of MYC addiction in T-ALL were transduced with PMSCV-luc-puro. Cells were counted and 2 million cells injected into NSG mice intravenously. Twenty-four hours after injection, mice were treated as indicated and imaged each day for 4 days to detect total flux by bioluminescence imaging. For bioluminescence imaging, animals were anesthetized with inhaled isoflurane and oxygen using the Xenogen XGI-8 5-port Gas Anesthesia System. d-luciferin was injected intraperitoneally (150 mg/kg) 10 min prior to imaging. Animals were placed into the light-tight chamber and were imaged with an IVIS-200 cooled CCD camera (Xenogen). Living Image (Xenogen) was used to collect and analyze data and generate pseudocolor images. Treatment groups were evaluated for statistical significance using Student’s *t* test. The murine RAS and BCR-ABL cell lines were generated previously from conditional murine transgenic models.

#### Subcutaneous experiments

NSG mice were also subcutaneously injected with BCR-ABL, RAS-dependent cell lines derived from conditional murine models. In addition, MYC-dependent Burkitt’s lymphoma cell line P493-6 was also subcutaneously injected into NSG mice with 10 × 10^6^ cells in PBS and treated as indicated. Tumors were measured every 2 days until tumor burden reached terminal endpoint, whereupon relevant tissue was collected.

### Oncogene inactivation

Conditional MYC-driven mouse T-ALL cell lines were derived from Em-tTA/tet-O-MYC mice1. c-MYC was inhibited in *Eμ-tTA/tet-O-MYC* T-ALL and P493-6 cells by treating cell cultures with 0.02 μg/ml doxycycline (Sigma-Aldrich, T7660) for indicated timepoints. The RAS and BCR-ABL cell lines were also generated from conditional murine transgenic models maintained by the Felsher laboratory (Stanford, School of Medicine). The conditional cell lines were confirmed to be negative for mycoplasma contamination and maintained in Roswell Park Memorial Institute 1640 medium (RPMI, Invitrogen) with GlutaMAX containing 10% fetal bovine serum, 100 IU/ml penicillin, 100 μg/ml streptomycin, and 50 mM 2-mercaptoethanol at 37 °C in a humidified incubator with 5% CO_2_.

### [U-^13^C]glucose tracing

The glucose tracing experiment was conducted as previously described [61]. In total, 1712 cells were treated with 20 ng/μl doxycycline, for 8 h, then changed to tracing medium containing 10 mM [U-13C]glucose for 24 h, with or without dox and TOFA at 3 μg/ml or 10 μg/ml. The tracing medium was removed by centrifugation at 200*g* for 5 min, and the cells were washed once with NaCl (0.9% w/v). Then, 1 ml 80% methanol was added to each tube containing the cell pellets. The samples were centrifuged at 20,000*g* for 15 min after 3 times frozen/thawed cycle. The supernatant was transferred and dried down for GC/MS analysis. For derivatization, 50 μl 10mg/ml methoxyamine hydrochloride (Sigma) was added to the dried samples and the samples were heated for 2 h at 42 °C, then 100 μl N-tert-Butyldimethylsilyl-N-methyltri fluoroacetamide (Sigma) was added and heated for 1.5 h at 72 °C. The supernatant was transferred for GC/MS analysis. The mass isotopomer abundances of palmitate were extracted with MATLAB (MathWorks, CA, USA). The mass isotopomer distribution (MID) of palmitate was calculated after natural isotope abundance correction. m + 2 isotopomer represents that 2 carbons of palmitate (totally 16 carbons) come from glucose, and the other isotopomers are the same. The atom percent of glucose-derived palmitate was calculated by the formula:

$$ \frac{\sum_{k=2}^n\left({p}_k\ast k\right)}{\left({\sum}_{k=2}^n{p}_k+{p}_o\right)\ast 16}\left(k=246810121416{p}_k\mathrm{represents}\ \mathrm{percent}\ \mathrm{of}\ m+k\ \mathrm{isotopomer}\right). $$

### Untargeted lipidomics

Lipid extraction was performed using methyl-tert-butyl ether (MBTE) [63]. Briefly, 1 × 10^6^ cells were lysed in ice-cold water with three freeze thaw cycles followed by addition of MBTE. The clear upper phase was collected; the lower phase was subjected to extraction of remaining lipids using additional MBTE. Both upper phases were combined and dried in a nitrogen evaporator (Organomation, Berlin, MA, USA). The dried extracts were reconstituted in isopropanol: acetonitrile (1:1, v/v) prior to analysis.

Untargeted lipidomics was performed using an Acclaim C30 reverse phase column (2.1 mm, 250 mm, 3.0 μm) on an RSLCnano (Thermo) system equipped with a high-flow HPG UPLC pump coupled to an Orbitrap Fusion Lumos tribrid mass spectrometer (Thermo Scientific, San Jose, CA). The mobile phases A (acetonitrile:water, 60:40 (v/v)) and B (isopropanol:acetontorile, 88:12, v/v), both containing 10 mM ammonium formate and 0.1% formic acid (v/v), were used for lipid separation. The flow rate was 0.3 mL/min with column temperature at 45 °C. We employed a 60 min gradient defined as follows: 30% B to 43% B for 0–4 min, 43–55 % B for 0.2 min, 55–65% B for 20 min, 65–85% B for 12 min, 85–100% B for 8 min, holding at 100% B for 7.8 min and stabilizing column at 30% B for 8 min. Data were acquired in positive/negative ion switching in a data-dependent analysis mode [78]. The data were subjected to identification, alignment, and relative quantitation using LipidSearch 4.1 (Thermo) with parameters described previously [78]. Relative lipid abundances were subjected to two-group comparisons. A t-test and fold-change analyses were performed to compare vehicle vs dox, vehicle vs T10, and vehicle vs T3. Multiple correction was applied using Benjamini-Hochberg and metabolites with adj. *p* value < 0.05 was considered as significant.

### Cell counting using Cytation5

#### Bright field only

Cell count was obtained using the Cytation5 with Biospa attachment (BioTek) by the following protocol: 3000 cells per well was plated in an Essen Bioscience ImageLock 96-well microplate (#4379) and let grow for 24 h prior to treatment. Wells were then treated with varying doses of TOFA (Cay10005263-1000, Cayman Chemical Company) or Firsocostat (S8893, Selleck Chemicals) at the following concentrations: .1, .3, 1, 3, 10, and 30 μg/ml. After treatment, the imaging protocol was created with the GEN5 application using bright field 4× images and applying a cell count analysis option to the image. Additionally, laser autofocus was set up to check the focus for each data point. The GEN5 protocol was then run on Biospa OnDemand software, capturing images and cell counts every 6 h for 4 days.

#### NucRed cells

Cell count was obtained using the Cytation5 with Biospa attachment (BioTek) by the following protocol: 3000 cells per well was plated in an Essen Bioscience ImageLock 96-well microplate (#4379) and let grow for 24 h prior to treatment. Wells were then treated with varying doses of TOFA (Cay10005263-1000, Cayman Chemical Company) or Firsocostat (S8893, Selleck Chemicals) at the following concentrations: .1, .3, 1, 3, 10, and 30 μg/ml. After treatment, the imaging protocol was created with the GEN5 application RFP 523 nm LED (PN 1225003, BioTek) images and applying a cell count analysis based on RFP signal to the image. Laser autofocus was set up to check the focus for each data point. The GEN5 protocol was then run on Biospa OnDemand software, capturing images and cell counts every 6 h for 4 days.

## Supplementary Information

**Additional file 1: Supplementary Figure 1.** Regulation of lipid pathway genes in RNA-seq and microarray experiments. A) Eμ-MYC model of B cell lymphoma RNA-seq data mining experiments focusing on the various expression profiles of multiple lipid synthesis pathways as the lymphoma progresses. Genes responsible for *de novo* fatty acid synthesis are striking in their increased expression patterns in B cell lymphoma progression driven by MYC (GSE 51011). B) RNA-seq was performed on cell lines (4188) derived from *Eμ-tTA/Tet-O-MYC* transgenic model in a MYC-off time-course. Decreased expression values were observed for multiple pathways such as glutaminolysis, which MYC is known to regulate. Sphingolipids also see reduced expression patterns which are one of the destinations for fatty acids after *de novo* lipid biogenesis. *De novo* fatty acid synthesis again seems to be strongly correlated after MYC expression is abrogated. (*data to be deposited in GEO upon publication*) after MYC expression is abrogated. C) Microarray analysis of BCR-ABL dependent lymphoma cell lines show that inhibition of BCR-ABL results in downregulation of key lipogenesis genes. Importantly, these genes appear to be linked to MYC downregulation with the exception of the BV173 cell line which has the highest MYC expression of the cell lines (GSE 23743). **Supplementary Figure 2.** Direct MYC biding at lipogenesis genes. A) ChIP-seq data shows direct MYC binding at promoters of the lipogenesis pathway upon increased MYC expression in Burkitt's like P493-6 cells (GSE 36354). B) ChIP-seq data in a conditional MYC expressing osteosarcoma cell line indicates that MYC-dependent regulation of lipogenesis is tissue dependent (GSE 44672). **Supplementary Figure 3.** MYC regulates lipogenesis genes. A) Cell lines (4188) derived from *Eμ-tTA/Tet-O-MYC* transgenic model were transduced with either empty vector (EMV) control, MYC overexpression vector (MYC), or the Miz1 non-binding mutant MYC-V394D (MYC-VD). Stable cell lines were generated and transgenic MYC was turned off for 24 hrs (dox) as seen in the EMV control MYC mRNA. MYC overexpression vector was not affected by doxycycline although the 4188 cells show decreased MYC due to loss of the transgenic MYC similar to EMV control levels. A similar expression pattern was observed for the MYC-VD binding mutant. For the MYC overexpression stable cell lines, there was no significant reduction in Acaca, Fasn, or Scd1, confirming MYC expression patterns influence these lipogenesis gene expression patterns. Interestingly the MYC-VD Miz1 binding mutant showed abrogation of mRNA expression similar to the EMV control suggesting that MYC/Miz1 directly influence Fasn, the committed step in *de novo* lipid biogenesis. These data confirmed doxycycline is not the agent responsible for reduced FA synthesis. B) The human cell line P493-6 showed similar expression profiles to the 4188 cells in panel A in the EMV control, but do not increased MYC expression with the additional overexpression vectors. ACACA, FASN, SCD, and MYC all show mRNA expression reduction in the EMV control. FASN and SCD did show significant changes in expression in the MYC overexpression with transgenic MYC off, although not to the levels observed in the EMV control. A similar pattern was observed for the Miz1 binding mutant. **Supplementary Figure 4.** Lymphoid malignant cells are sensitive to fatty acid inhibition. A) We obtained human cell lines derived from hematologic malignancies and driven by different oncogenes (as indicated). We also tested 3 ACACA inhibitors (TOFA, firsocostat, CP-640186) and one FASN inhibitor (TVB-2640) and dosed the cells for 24 (data not shown) and 48 hr timepoints. With the exception of firsocostat all *de novo* lipogenesis inhibitors reduced viable cell populations, suggesting that inhibition of FA synthesis may be a metabolic liability in blood cancers. Similar data was obtained at the 24 hr timepoints, although we chose to show the 48 hr time point due to the lack of efficacy in the firsocostat treatment group. B) Ramos cells were transduced with virus generated from the LentiCRISPRV2 (Addgene #52961) with three sgRNA FASN guides from the GeCKO library cloned into the vector. The pooled virus were used to transduce the RAMOS cells, and we observed resistance to TOFA treatment between the range of no-effect to complete cell death (panel A). mRNA confirmation of FASN expression show a 63% reduction compared to empty vector control. *P⩽0.1; **P⩽0.01; **P⩽0.001; ***P⩽0.001; ****P⩽0.0001. **Supplementary Figure 5.** Fatty acid inhibition in human colon, pancreatic, and hepatoblastoma cancer cell lines. Various cell lines were subjected to the same dose response as the lymphoid cells in Supplementary Fig. 4A. These adherent cells were monitored using a Cytation5 every 6-8 hrs for 4 days. Gemcitabine control was dosed at 1 nM. A) Three colon cancer cell lines tested for TOFA dose response: DLD1 (MYC), HCT116 (CDKN2B), RKO (BRAF). These cells are moderately sensitive to prolonged TOFA treatment at high concentrations. None of the cell lines tested achieved an ED50 within the 48 hr timepoint as compared to lymphoid cells (Supplementary Figure 4A). B) Three Pancreatic cancer cell lines were tested for dose response: HPAFII (KRAS), MIA PaCa-2 (KRAS), PANC-1 (KRAS). Both HPAFII and MIA PaCa-2 cell lines achieved ED50 between 48 and 54 hrs, while the PANC1 cell line displayed moderate sensitivity to TOFA treatment. C) Hepatoblastoma cell line HepG2 (chromosomal amplified) cells are largely resistant to TOFA treatment. **Supplementary Figure 6.** Oncogene modulation and response to TOFA. A) Expanded data from Fig. 4A. TOFA treatment for 24 and 48 hr in either MYC, RAS, or BCR-ABL-dependent lymphoid in the additional oncogene on vs off results in significant increased cell death as detected by Annexin V and 7-AAD (TOFA 5.5 μg/ml). Summary tables are included to the right of the panel. A) For MYC, the loss of the oncogene reduces the sensitivity to TOFA, suggesting transcriptional influences in active gene programs. B-C) In BCR-ABL and RAS cell lines the loss of the oncogene contributes to a 10% increase in Annexin V staining, suggesting an increased dependency on the oncogenic perturbation. **Supplementary Figure 7.** TOFA treatment results in apoptosis of T-ALL cells. A) NSG mice were intravenously injected with MYC-driven T-ALL cells derived from *Eμ-tTA/Tet-O-MYC* transgenic model, and treated with TOFA for 4 days on, followed by 4 days off and were then sacrificed. Cleaved caspase 3 is significantly more prevalent in splenic tissue from TOFA treated mice when compared to control and is associated with the T cell marker CD4. B) Due to the slight background observed in vehicle samples, we included a no secondary control for comparison, and confirmation that the background is not due to autofluorescence. **Supplementary Figure 8.** Unfolded protein response stress. UPR stress response genes were monitored for changes in expression levels in response to lipogenesis blockade in MY, BCR-ABL, and RAS-dependent cell lines. Cells were treated with TOFA for 24 hrs and expression levels of the indicated lipogenesis genes were monitored. Although UPR stress responses were modulated in each cell line, the expression levels do not appear to be related to the observed increase in lipogenesis. B) Cell lines derived from *Eμ-tTA/Tet-O-MYC* model were injected intravenously into NOD-SCIDIL-2Rg^-/-^ and tracked for engraftment. Splenic tissue was collected from moribund treated with either vehicle or TOFA as indicated, and mRNA levels were monitored. Comparisons to vehicle control utilized unpaired *t*-test **P⩽0.01; **P⩽0.001; ***P⩽0.001; ****P⩽0.0001. **Supplementary Figure 9.** Response to TOFA in human cell lines. A) A panel of human lymphoid cell lines were treated with increasing doses of TOFA and cell populations were monitored (proliferation) via metabolic activity (CellTiter-Glo assay, Promega). ED50s were calculated with the exception of the non-responsive cells. Dose escalation was not possible due to the potential effects attributable to vehicle (DMSO) toxicity. **Supplementary Figure 10.** Oncogene expression profiles in a panel of lymphoid malignancies. A) We obtained a panel of human lymphoma cells and measured relative MYC, RAS, and ABL1 expression levels. Cells with low expression of target result in high error bars, a common issue for qRT-PCR experiments. Median expression value is indicated by dotted line.

## Data Availability

The datasets used and analyzed during the current study are available on the central repository GEO: GSE 51011, GSE 23743, GSE 36354, GSE 44672. The RNA-seq time-course data utilized in Fig. 1C and supplementary Fig. 1B are available in GEO: GSE178580. Data are available from the corresponding author upon written request.

## Abbreviations

MYC: c-MYC
ER stress: Endoplasmic reticulum stress
UPR stress: Unfolded protein response stress
ACACA (or ACC): Acetyl-CoA carboxylase
NSG: NOD-SCID IL-2Rg^−/−^ mice
TOFA: 5-(Tetradecloxy)-2-furic acid
MYC ON: No doxycycline
MYC OFF: 20 ng/ml doxycycline (same for RAS and BRC-ABL and P493-6)
FASN: Fatty acid synthase
IV: Intravenous
BLI: Bioluminescence imaging
FA: Fatty acid
SCD: Stearoyl-CoA desaturase
DOX: Doxycycline
MG: Monoglycerides
DG: Diglycerides
TG: Triglycerides
PCs: Phosphatidylcholines
PE: Phosphatidylethanolamines
CC3: Cleaved caspase-3
ConA: Concanavalin A
CHOP: C/EBP homologous protein
LysoPCs: Lysophosphatidylethanolamines
