# Supplementary material for: Metabolic convergence on lipogenesis in RAS, BCR-ABL, and MYC-driven lymphoid malignancies
Source: Cancer Metab. 2021 Aug 16;9:31. doi: 10.1186/s40170-021-00263-8 (PMC8369789; doi:10.1186/s40170-021-00263-8)
Supplement: Supplementary file 1 — Additional file 1: Supplementary Figure 1. Regulation of lipid pathway genes in RNA-seq and microarray experiments. A) Eμ-MYC model of B cell lymphoma RNA-seq data mining experiments focusing on the various expression profiles of multiple lipid synthesis pathways as the lymphoma progresses. Genes responsible for de novo fatty acid synthesis are striking in their increased expression patterns in B cell lymphoma progression driven by MYC (GSE 51011). B) RNA-seq was performed on cell lines (4188) derived from Eμ-tTA/Tet-O-MYC transgenic model in a MYC-off time-course. Decreased expression values were observed for multiple pathways such as glutaminolysis, which MYC is known to regulate. Sphingolipids also see reduced expression patterns which are one of the destinations for fatty acids after de novo lipid biogenesis. De novo fatty acid synthesis again seems to be strongly correlated after MYC expression is abrogated. (data to be deposited in GEO upon publication) after MYC expression is abrogated. C) Microarray analysis of BCR-ABL dependent lymphoma cell lines show that inhibition of BCR-ABL results in downregulation of key lipogenesis genes. Importantly, these genes appear to be linked to MYC downregulation with the exception of the BV173 cell line which has the highest MYC expression of the cell lines (GSE 23743). Supplementary Figure 2. Direct MYC biding at lipogenesis genes. A) ChIP-seq data shows direct MYC binding at promoters of the lipogenesis pathway upon increased MYC expression in Burkitt's like P493-6 cells (GSE 36354). B) ChIP-seq data in a conditional MYC expressing osteosarcoma cell line indicates that MYC-dependent regulation of lipogenesis is tissue dependent (GSE 44672). Supplementary Figure 3. MYC regulates lipogenesis genes. A) Cell lines (4188) derived from Eμ-tTA/Tet-O-MYC transgenic model were transduced with either empty vector (EMV) control, MYC overexpression vector (MYC), or the Miz1 non-binding mutant MYC-V394D (MYC-VD). Stable cell lines wer [file 40170_2021_263_MOESM1_ESM.docx]

**Supplementary Figure legends**

**Supplementary figure 1. Regulation of lipid pathway genes in RNA-seq and microarray experiments.** A) Eµ-MYC model of B-cell lymphoma RNA-seq data mining experiments focusing on the various expression profiles of multiple lipid synthesis pathways as the lymphoma progresses. Genes responsible for *de novo* fatty acid synthesis are striking in their increased expression patterns in B-cell lymphoma progression driven by MYC (GSE 51011). B) RNA-seq was performed on cell lines (4188) derived from *Eµ-tTA/Tet-O-MYC* transgenic model in a MYC-off time-course. Decreased expression values were observed for multiple pathways such as glutaminolysis, which MYC is known to regulate. Sphingolipids also see reduced expression patterns which are one of the destinations for fatty acids after *de novo* lipid biogenesis. *De novo* fatty acid synthesis again seems to be strongly correlated after MYC expression is abrogated. (*data to be deposited in GEO upon publication*) after MYC expression is abrogated. C) Microarray analysis of BCR-ABL dependent lymphoma cell lines show that inhibition of BCR-ABL results in downregulation of key lipogenesis genes. Importantly, these genes appear to be linked to MYC downregulation with the exception of the BV173 cell line which has the highest MYC expression of the cell lines (GSE 23743)

**Supplementary figure 2. Direct MYC biding at lipogenesis genes.** A) ChIP-seq data shows direct MYC binding at promoters of the lipogenesis pathway upon increased MYC expression in Burkitt's like P493-6 cells (GSE 36354). B) ChIP-seq data in a conditional MYC expressing osteosarcoma cell line indicates that MYC-dependent regulation of lipogenesis is tissue dependent (GSE 44672).

**Supplementary figure 3. MYC regulates lipogenesis genes.** A) Cell lines (4188) derived from *Eµ-tTA/Tet-O-MYC* transgenic model were transduced with either empty vector (EMV) control, MYC overexpression vector (MYC), or the Miz1 non-binding mutant MYC-V394D (MYC-VD). Stable cell lines were generated and transgenic MYC was turned off for 24hrs (dox) as seen in the EMV control MYC mRNA. MYC overexpression vector was not affected by doxycycline although the 4188 cells show decreased MYC due to loss of the transgenic MYC similar to EMV control levels. A similar expression pattern was observed for the MYC-VD binding mutant. For the MYC overexpression stable cell lines, there was no significant reduction in Acaca, Fasn, or Scd1, confirming MYC expression patterns influence these lipogenesis gene expression patterns. Interestingly the MYC-VD Miz1 binding mutant showed abrogation of mRNA expression similar to the EMV control suggesting that MYC/Miz1 directly influence Fasn, the committed step in *de novo* lipid biogenesis. These data confirmed doxycycline is not the agent responsible for reduced FA synthesis. B) The human cell line P493-6 showed similar expression profiles to the 4188 cells in panel A in the EMV control, but do not increased MYC expression with the additional overexpression vectors. ACACA, FASN, SCD, and MYC all show mRNA expression reduction in the EMV control. FASN and SCD did show significant changes in expression in the MYC overexpression with transgenic MYC off, although not to the levels observed in the EMV control. A similar pattern was observed for the Miz1 binding mutant.

**Supplementary figure 4. Lymphoid malignant cells are sensitive to fatty acid inhibition.** A) We obtained human cell lines derived from hematologic malignancies and driven by different oncogenes (as indicated). We also tested 3 ACACA inhibitors (TOFA, firsocostat, CP-640186) and one FASN inhibitor (TVB-2640) and dosed the cells for 24 (data not shown) and 48hr timepoints. With the exception of firsocostat all *de novo* lipogenesis inhibitors reduced viable cell populations, suggesting that inhibition of FA synthesis may be a metabolic liability in blood cancers. Similar data was obtained at the 24hr timepoints, although we chose to show the 48hr time point due to the lack of efficacy in the firsocostat treatment group. B) Ramos cells were transduced with virus generated from the LentiCRISPRV2 (Addgene #52961) with three sgRNA FASN guides from the GeCKO library cloned into the vector. The pooled virus were used to transduce the RAMOS cells, and we observed resistance to TOFA treatment between the range of no-effect to complete cell death (panel A). mRNA confirmation of FASN expression show a 63% reduction compared to empty vector control. *P⩽0.1; **P⩽0.01; **P⩽0.001; ***P⩽0.001; ****P⩽0.0001.

**Supplementary figure 5. Fatty acid inhibition in human colon, pancreatic, and hepatoblastoma cancer cell lines**. Various cell lines were subjected to the same dose response as the lymphoid cells in Supplementary Fig. 4A. These adherent cells were monitored using a Cytation5 every 6-8hrs for 4 days. Gemcitabine control was dosed at 1nM. A) Three colon cancer cell lines tested for TOFA dose response: DLD1 (MYC), HCT116 (CDKN2B), RKO (BRAF). These cells are moderately sensitive to prolonged TOFA treatment at high concentrations. None of the cell lines tested achieved an ED50 within the 48hr timepoint as compared to lymphoid cells (Supplementary Figure 4A). B) Three Pancreatic cancer cell lines were tested for dose response: HPAFII (KRAS), MIA PaCa-2 (KRAS), PANC-1 (KRAS). Both HPAFII and MIA PaCa-2 cell lines achieved ED50 between 48-54hrs, while the PANC1 cell line displayed moderate sensitivity to TOFA treatment. C) Hepatoblastoma cell line HepG2 (chromosomal amplified) cells are largely resistant to TOFA treatment.

**Supplementary figure 6. Oncogene modulation and response to TOFA.** A) Expanded data from Fig 4A. TOFA treatment for 24 and 48hr in either MYC, RAS, or BCR-ABL-dependent lymphoid in the additional oncogene on vs off results in significant increased cell death as detected by Annexin V and 7-AAD (TOFA 5.5 µg/ml). Summary tables are included to the right of the panel. A) For MYC, the loss of the oncogene reduces the sensitivity to TOFA, suggesting transcriptional influences in active gene programs. B-C) In BCR-ABL and RAS cell lines the loss of the oncogene contributes to a 10% increase in Annexin V staining, suggesting an increased dependency on the oncogenic perturbation.

**Supplementary figure 7. TOFA treatment results in apoptosis of T-ALL cells**. A) NSG mice were intravenously injected with MYC-driven T-ALL cells derived from *Eµ-tTA/Tet-O-MYC* transgenic model, and treated with TOFA for 4 days on, followed by 4 days off and were then sacrificed. Cleaved caspase 3 is significantly more prevalent in splenic tissue from TOFA treated mice when compared to control and is associated with the T-cell marker CD4. B) Due to the slight background observed in vehicle samples, we included a no secondary control for comparison, and confirmation that the background is not due to autofluorescence.

**Supplementary figure 8. Unfolded protein response stress.** UPR stress response genes were monitored for changes in expression levels in response to lipogenesis blockade in MY, BCR-ABL, and RAS-dependent cell lines. Cells were treated with TOFA for 24hrs and expression levels of the indicated lipogenesis genes were monitored. Although UPR stress responses were modulated in each cell line, the expression levels do not appear to be related to the observed increase in lipogenesis. B) Cell lines derived from *Eµ-tTA/Tet-O-MYC* model were injected intravenously into NOD-SCIDIL-2Rg^-/-^ and tracked for engraftment. Splenic tissue was collected from moribund treated with either vehicle or TOFA as indicated, and mRNA levels were monitored. Comparisons to vehicle control utilized unpaired T test **P⩽0.01; **P⩽0.001; ***P⩽0.001; ****P⩽0.0001.

**Supplementary figure 9. Response to TOFA in human cell lines.** A) A panel of human lymphoid cell lines were treated with increasing doses of TOFA and cell populations were monitored (proliferation) via metabolic activity (CellTiter Glo assay, Promega). ED50s were calculated with the exception of the non-responsive cells. Dose escalation was not possible due to the potential effects attributable to vehicle (DMSO) toxicity.

**Supplementary Figure 10: Oncogene expression profiles in a panel of lymphoid malignancies.** A) We obtained a panel of human lymphoma cells and measured relative MYC, RAS, and ABL1 expression levels. Cells with low expression of target result in high error bars, a common issue for qRT-PCR experiments. Median expression value is indicated by dotted line.
